# 

**Published:** 1907-06

**Authors:** 


### BOOKS RECEIVED.

Chemical Pathology. Being a Discussion of General Pathology from the Standpoint of the Chemical Processes Involved. By H. Gideon Wells, Ph.D., M.D., Assistant Professor of Pathology in the University of Chicago and in Rush Medical College, Chicago. Octavo of 549 pages. Philadelphia and London: W. B. Saunders Company, 1907. (Cloth, $3.25 net.)

---

A Textbook of Embryology. By John C. Heisler, M.D., professor of Anatomy in the Medico-Chirurgical College of Philadelphia. Third revised edition. Octavo volume of 432 pages, with 212 illustrations, 32 of them in colors. Philadelphia and London: W. B. Saunders Company, 1907. (Cloth, $3.00 net; half morocco, $4.25 net.)

---

Diagnostics of Diseases of Children. By LeGrand Kerr, M.D., Professor of Diseases of Children at the Brooklyn Postgraduate Medical School. Octavo of 542 pages, illustrated. Philadelphia and London: W. B. Saunders Company, 1907. (Cloth, $5.00 net; half morocco, $6.50 net.)

---

Surgical Diagnosis. By Daniel N. Eisendrath, M.D., Adjunct Professor of Surgery in the Medical Department of the University of Illinois (College of Physicians and Surgeons). Octavo of 775 pages, with 482 original illustrations, 15 in colors. Philadelphia and London: W. B. Saunders Company, 1907. (Cloth, $6.50 net; half morocco, $8.00 net.)

Modern Surgery: General and Operative. By J. Chalmers DaCosta, M.D., Professor of the Principles of Surgery and of Clinical Surgery in the Jefferson Medical College, Philadelphia. Fifth revised edition. Enlarged and reset. Octavo volume of 1283 pages, with 872 illustrations, some in colors. Philadelphia and London: W. B. Saunders Company, 1907. (Cloth, $5.50 net; half morocco, $7.00 net.)

---

The American Pocket Medical Dictionary. Edited by W. A. Newman Dorland, M.D., editor "The American Illustrated Medical Dictionary." Fifth revised edition. 32mo of 574 pages. Philadelphia and London: W. B. Saunders Company, 1906. Flexible morocco, gold edges, $1.00 net; thumb indexed, $1.25 net.)

---

Atlas and Epitome of Diseases of Children. By Dr. R. Hecker and Dr. J. Trumpp, of Munich. Edited, with additions, by Isaac A. Abt, M.D., Assistant Professor of the Diseases of Children in Rush Medical College, in affiliation with the University of Chicago. With 48 colored plates, 147 black and white illustrations, and 453 pages of text. Philadelphia and London: W. B. Saunders Company, 1907. Cloth, $5.00 net.

---

A Manual of Personal Hygiene: Proper Living upon a Physiologic Basis. By eminent specialists. Edited by Walter L. Pyle, M.D., Assistant Surgeon to the Wills Eye Hospital, Philadelphia. Third revised edition. 12mo of 451 pages, illustrated. Philadelphia and London: W. B. Saunders Company, 1907. (Cloth, $1.50 net.)

---

A Manual of the Diagnosis and Treatment of the Diseases of the Eye. By Edward Jackson, M.D., Professor of Ophthalmology in the University of Colorado. Second revised edition. 12mo of 615 pages with 182 text-illustrations and 2 colored plates. Philadelphia and London: W. B. Saunders Company, 1907. (Cloth, $2.50 net.)

---

A Textbook of Materia Medica for Nurses. Including Therapeutics and Toxicology. By George P. Paul, M.D., Assistant Visiting Physician and Adjunct Radiographer to the Samaritan Hospital, Troy, N. Y. 12mo of 240 pages. Philadelphia and London: W. B. Saunders Company, 1907. (Cloth, $1.50 net.)

---

The Care of the Baby. By J. P. Crozer Griffith, M.D., Clinical Professor of Diseases of Children in the Hospital of the University of Pennsylvania. Fourth revised edition. 12mo of 455 pages illustrated. Philadelphia and London: W. B. Saunders Company, 1907. (Cloth, $1.50 net.)

---

The Essentials of Histology, Descriptive and Practical. For the use of Students. By Edward A. Schafer, F.R.S., Professor of Physiology in University College, London. New (7th) edition, revised and enlarged. Octavo, 507 pages, with 552 illustrations. Lea Brothers and Co., Philadelphia and New York, 1907. (Cloth, $3.50, net).

---

A Treatise on the Practice of Medicine. For Practitioners and Students. By Arthur R. Edwards, M.D., Professor of the Principles and Practice of Medicine and Clinical Medicine in the Northwestern University Medical School, Chicago. Octavo, 1328 pages, with 101 engravings and 19 plates. Lea Brothers & Co., Philadelphia and New York, 1907. (Cloth, $5.50, net; leather, $6.50, net).

---

Modern Medicine. Its Theory and Practice. In Original Contributions by American and Foreign Authors. Edited by William

Osler, M.D., Regius Professor of Medicine in Oxford University, England; formerly Professor of Medicine in Johns Hopkins University, Baltimore; in the University of Pennsylvania, Philadelphia, and in McGill University, Montreal. Assisted by Thomas McCrea, M.D., Associate Professor of Medicine and Clinical Therapeutics in Johns Hopkins University, Beltimore. In seven octavo volumes of about 1,000 pages each; illustrated. Volume I. Lea Brothers & Co., Philadelphia and New York, 1907-1908. (Price per volume, cloth, $6.00; leather, $7.00; half morocco, $7.50, net prices).

---

A Practician's Hand-Book of Materia Medica and Therapeutics, based upon established physiologic actions and the indications in small doses. By Thomas S. Blair, M.D. 250 pages, net. Published by The Medical Council, 4105 Walnut street, Philadelphia, Pa. (Price $2.00.)

---

Medical Jurisprudence, Forensic Medicine, and Toxiocology. By R. A. Witthaus, A.M., M.D., Professor of Chemistry, Physics and Toxiocology in Cornell University; and Tracy C. Becker, A.B., LL.B., Professor of Criminal Law and Medical Jurisprudence in the University of Buffalo. Second edition. Vol. II. Octavo, pp. vii-1008. New York: William Wood & Co. 1907. (Muslin, $6.00; law sheep, $7.00, net prices).

---

Transaction of the twelfth annual meeting of the American Laryngological, Rhinological and Otological society held at Kansas City, Mo., June 11-12, 1906. Wendell C. Phillips, M.D., secretary.

---

Wellcome's photographic exposure. Record and diary, 1907. Burroughs Wellcome and Co., 45, Lafayette St., New York.

